# Physiological and immunological aspects of asthma: the guinea pig (*Cavia porcellus*) as a model of allergic asthma

**DOI:** 10.1186/s42826-025-00253-7

**Published:** 2025-09-22

**Authors:** Ivonne Pacheco-Alba, Marisol Alvarez-González

**Affiliations:** Laboratorio de Inmunofarmacología, Instituto Nacional de Enfermedades Respiratorias Ismael Cosio Villegas, Mexico City, Mexico

**Keywords:** Allergen, Animal models, Airways, Bronco-obstruction, Pathophysiology, Hyperresponsiveness, Inflammation, Contraction, Smooth muscle

## Abstract

Asthma is a chronic and heterogeneous airway disease characterized by a variety of respiratory symptoms associated with airflow limitation. Asthma patients exhibit altered immunological and physiological features in the airways, including inflammation, hyperresponsiveness, and, in severe cases, permanent structural changes that lead to airway obstruction. Among the different types of asthma, allergic asthma mediated by Th2 cells is the most prevalent phenotype worldwide. The diversity of etiological factors involved, the variability in symptom intensity, and the high global incidence have increased interest in studying this phenomenon. Due to the ethical constraints associated with studying asthma in humans, the development of animal models has emerged as an alternative for investigating the disease’s pathophysiology. In particular, the guinea pig (*Cavia porcellus*) has become one of the most commonly used species, as it closely resembles the inflammatory, pharmacological, and physiological responses observed in the human airway. This article provides a comprehensive description of the development of an allergic asthma model in the guinea pig. The processes involved in each methodological phase are described in detail from an immunological and physiological perspective, emphasizing their importance in understanding the disease’s pathophysiological mechanisms. It is argued that the airway inflammation, obstructive responses, and remodeling processes observed in this model are consistent with features seen in asthma patients, establishing the guinea pig as a reliable model for studying allergic asthma in humans.

## Background

Asthma is a complex disease characterized by inflammation and obstruction of the airways, leading to a series of physiological processes associated with clinical symptoms such as wheezing, shortness of breath, chest tightness, and coughing [1]. According to the World Health Organization, as of 2019, the disease affected 262 million people globally and was responsible for approximately 461,000 deaths [2]. In Mexico, 8.5 million individuals suffer from asthma, a condition that, prior to the COVID-19 pandemic, had an incidence rate ranging from 193.14 to 201.23 cases per 100,000 inhabitants [3].

The heterogeneity of asthma is usually attributed to the interaction of environmental factors, genetic factors, and comorbidities in affected individuals [4]. Therefore, the classification of asthma is crucial for both research and treatment purposes. Currently, asthma is categorized into phenotypes, based on symptoms and clinical history, and endotypes, defined by the predominant type of inflammatory cells in each individual [5]. Asthma manifests in a broad spectrum of phenotypes related to immune responses and triggers of airway obstruction, such as allergic asthma, non-allergic asthma, late-onset asthma, persistent asthma, and obesity-associated asthma. However, the most common phenotype worldwide is allergic asthma [1, 6].

Allergic asthma is characterized by "allergic sensitization," a phenomenon triggered by exposure to typically harmless environmental allergens, which elicit an exaggerated and dysregulated immune-inflammatory response in predisposed individuals [7]. This inflammatory response in the airways is primarily mediated by T helper type 2 (Th2) lymphocytes and proinflammatory cytokines associated with this immune response [8]. Interleukins (IL)−4, IL-5 and IL-13, together with immunoglobulin E (IgE), mast cells, and eosinophils, play a central role in the pathophysiology of asthma by exacerbating inflammation and inducing variable and reversible bronchoconstriction in the airway [9, 10]. This inflammatory process enhances excessive bronchoconstriction, a key factor in the progression of the disease [11, 12].

In patients with chronic and poorly controlled asthma, persistent and prolonged inflammation can lead to permanent structural changes in the airways. These changes, characterized by alterations in the composition, organization and structural function of cells and the surrounding extracellular matrix, lead to a phenomenon known as airway remodeling [13–15]. In addition to inflammation, intrinsic properties of airway smooth muscle also contribute significantly to the exacerbation of contractile spasm, leading to a phenomenon known as airway hyperresponsiveness (AHR) [16].

In this context, the study of the pathophysiological and pharmacological aspects of asthma in humans is confronted with various ethical problems that limit the progress of research. As an alternative, animal models have proven to be an effective tool to reproduce important aspects of the disease [17, 18]. Therefore, this review article aims to describe in detail the processes involved in the development of the allergic asthma model in guinea pigs, one of the most efficient species to study this pathology. Furthermore, the relevance of using this model is addressed, highlighting the immunological and physiological parameters that can be measured and analyzed at each methodological stage, with the aim of deepening the understanding of the underlying mechanisms of the disease.

## Main text

### The guinea pig (Cavia porcellus) as a model allergic asthma

Each allergic asthma model developed in different animal species aims to mimic different features of the disease, including Th2 cell-mediated inflammation, airway obstruction, AHR and, in some cases, airway remodeling. These models have provided valuable insights into the pathophysiologic and pharmacologic processes underlying the disease [17]. However, they exhibit inherent limitations that prevent replication of all disease characteristics in a single animal model [18, 19]. Currently, rodents are the most commonly used option for allergic asthma research as they are inexpensive and easy to handle in the laboratory, with mice and guinea pigs being the most commonly used species [20].

Mouse models of allergic asthma have facilitated the study of several features that are very similar to those of human disease, including the presence of allergic markers such as IgE, inflammation and AHR. In addition, due to the wide availability of commercial reagents and transgenic animals, analysing samples from mice is relatively easy and allows a more detailed understanding of the underlying pathophysiological mechanisms. However, certain physiological limitations, such as lack of late-phase bronchoconstriction, lack of chronic response to allergens and immunological tolerance, limit the utility of mouse models for allergic asthma research [21]. Guinea pigs, on the other hand, were among the first species to be used as experimental models of asthma, and their physiological and immunological characteristics continue to make them highly relevant, as they present fewer limitations compared to mice. In particular, guinea pigs show late-phase bronchoconstriction and a chronic response to allergens, both of which are crucial for a comprehensive study of the disease [20, 22].

Compared to mice, the anatomy of the respiratory tract of guinea pigs is more similar to that of humans. Tissue structures such as pseudostratified epithelium, direct neural innervation in the epithelium and subepithelial spaces, autonomic innervation in the airway smooth muscle, subepithelial vasculature and the abundance of goblet cells in the airways allow a more comprehensive study of asthma in guinea pigs. In contrast, these characteristics are less pronounced in mice. They lack subepithelial vasculature and present an airway epithelium with limited innervation [23]. Furthermore, the anatomy and physiology of guinea pig airway smooth muscle is very similar to that of humans, facilitating its use in pharmacologic and treatment studies. This similarity results from the high homology of receptor expression in the airways of guinea pigs and humans, as well as the comparable response to contractile and relaxant agonists, which exhibit nearly identical potency and efficacy in both species [23]. In contrast, mice present a smaller amount of smooth muscle in the airway, and the variability of key contraction triggers, such as serotonin in mice but not in humans, makes it difficult to extrapolate these models to human physiology [24].

With regard to the inflammatory response, previous studies have shown that the allergic response in guinea pigs is associated with an increase in the production of key inflammatory mediators, including IgE, IL-4, IL-5, IL-6, IL-8, IL-13, IL-17 and tumor necrosis factor alpha (TNF-α), similar to humans [25–27]. In addition, increased infiltration of eosinophils, neutrophils and mast cells has been observed [25, 28], which are characteristic cells of allergic inflammation. At the same time, increased production of secondary mediators crucial for the development of bronchospasm, such as cysteinyl leukotrienes (CysL), platelet-activating factor (PAF) and eotaxin, infiltrates the airway wall, a process analogous to that observed in humans [23, 29]. In contrast, although mouse models show Th2 inflammatory responses similar to allergic responses in humans, including IL-4, IL-9, IL-13, transforming growth factor-beta (TGF-β), and increased eosinophils, the expression of these inflammatory components varies considerably depending on the strain used, making the collective assessment of inflammatory mediators and the regulation of their signaling pathways difficult [30].

Finally, the guinea pig asthma model offers significant advantages in the study of structural changes in the airways, as it allows detailed analysis of components such as mucus secretion, cell proliferation and remodeling, protein expression levels, and second messengers in airway smooth muscle involved in various signaling pathways critical for asthma development [31, 32]. These changes can modify the airway environment, influence tissue development and alter both the mechanical behavior and the inflammatory response of the airways [20].

To ensure reproducibility and accuracy in simulating the characteristics of allergic asthma, certain protocols must be followed when developing a guinea pig model. These protocols are divided into four phases: allergic sensitization, antigen reinforcement, antigen challenges and analysis of AHR. Once allergic inflammation is established in the model, the animals are exposed to the antigen at regular intervals, a procedure called "antigen challenge" that mimics the exacerbations of asthma patients [31, 33].

### Allergic sensitization

Sensitization is the initial step in the development of the allergic asthma model. It involves the first exposure of guinea pigs to the allergen, which triggers the Th2-mediated allergic inflammatory response and the production of antigen-specific IgE antibodies [34, 35]. Since most animals do not naturally develop asthma [18], it must be induced through exposure to allergens and adjuvants that stimulate the immune system. To this end, a wide range of allergens in animal asthma models. These include species of house dust mites such as *Dermatophagoides pteronyssinus* and *Dermatophagoides farinae* [21, 36], certain fungal species such as *Aspergillus fumigatus*, cockroach extracts or even some bacteria such as *Bordetella pertussis* [37]. However, ovalbumin (OVA) is commonly used due to its efficient ability to induce allergic lung inflammation in laboratory rodents [38].

The use of solutions containing OVA as antigen and aluminum hydroxide (Al(OH)₃) as adjuvant, administered subcutaneously or intraperitoneally, is a well-established method to induce an allergic inflammatory response by a minimally invasive approach [39]. The choice of the route of administration for OVA is crucial for the development of the inflammatory response, as both the subcutaneous and intraperitoneal regions facilitate the uptake of the antigen due to their high concentration of leukocytes and dendritic cells (DCs), which resemble lymphoid tissue [40–42]. Furthermore, the use of two routes of administration is closely related to the intensity of the observed response in guinea pigs. It has been documented that exclusive intraperitoneal administration increases mortality in guinea pigs due to direct inoculation of OVA into the systemic circulation [43, 44], resulting in an exacerbated allergenic response. In contrast, subcutaneous administration promotes a slower and more controlled diffusion of OVA throughout the organism [45].

**Fig. 1:**
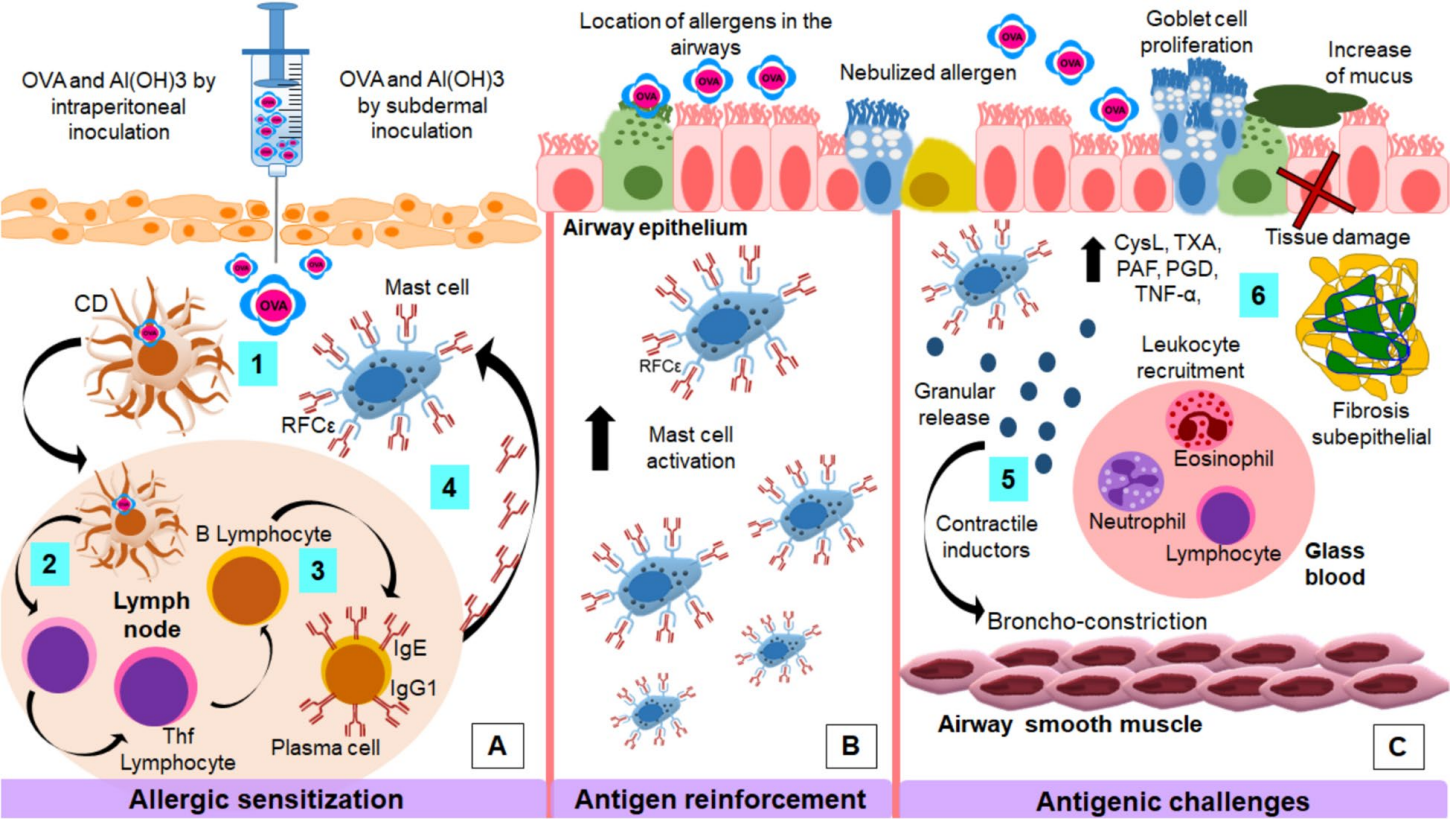
Allergic asthma model in guinea pigs. Antigen sensitization. 1. Intraperitoneal and subcutaneous administration of the OVA/Al(OH)₃ solution enhances antigen uptake and processing by DCs. 2. DCs migrate to the lymph nodes, where they activate naïve Th0 lymphocytes, leading to their differentiation into Tfh and Th2 lymphocytes. 3. Tfh lymphocytes promote the differentiation of B lymphocytes into plasma cells, which secrete IgE and IgG1 antibodies. 4. IgE binds to its high-affinity FcεR receptors on mast cells, priming them for antigen recognition. Antigen reinforcement. Re-exposure to the nebulized allergen leads to localized mast cell in the airways. Antigen challenges. 5. Nebulized OVA exposure during antigen challenges induces mast cell degranulation, releasing various contractile mediators that cause airway smooth muscle contraction. 6. This process results in increased production of pro-inflammatory mediators, goblet cell proliferation, mucus hypersecretion, lymphocyte recruitment, epithelial damage, and subepithelial fibrosis, particularly in models of persistent or chronic asthma

From an immunological perspective, during allergic sensitization to the OVA, DCs capture and process the allergen (OVA) and migrate to the lymph nodes where they present the antigen to naïve Th0 lymphocytes [46, 47]. Antigen presentation is mediated by the interaction between the major histocompatibility complex class II (MHC-II) receptor on DCs and the T cell receptor (TCR) on lymphocytes [48]. To ensure lymphocyte activation, DCs express costimulatory receptors and molecules such as CD40 and CD80, which bind to their respective ligands, CD40L and CD28, on lymphocytes [49, 50]. Once activated, Th0 lymphocytes differentiate into follicular T helper (Tfh) and Th2 cells, a process driven by increased IL-4 production in the microenvironment in response to antigen presentation [51, 52]. This phenomenon occurs on a large scale and triggers a systemic immune response against OVA (Fig. 1A) and is usually enhanced by the administration of Al(OH)₃ as an adjuvant, which directly modulates the immune system by promoting the polarization of the Th2 response [39, 53].

In the lymph nodes, activated Tfh lymphocytes increase the production of IL-13 and IL-4 and thus promote the differentiation of B lymphocytes into plasma cells. These plasma cells synthesize specific antibodies against OVA, predominantly of the IgE isotype, as well as IgG1 in guinea pigs [54–56]. The circulating IgE binds to its high-affinity FcɛRI receptors, which are located on the cell membranes of mast cells and basophils. The fixation of IgE on the surface of these cells, a process known as "sensitization", marks the onset of an allergic response to the antigen [57, 58]. In this experimental model, sensitization represents the establishment of an OVA allergy that enables the previously sensitized mast cells to trigger specific allergic responses upon subsequent exposures to the antigen.

### Antigen reinforcement

Antigen reinforcement represents the second phase of the allergic asthma model, in which guinea pigs are re-exposed to the previously sensitized allergen, usually by nebulization [33, 56]. Little is known about the immunological processes that occur during this phase in guinea pig asthma models; however, it is essential for the subsequent responses in the model. In this context, studies by Bazán-Perkins [33] and Álvarez-Santos [59] have shown that guinea pigs that received only the antigenic reinforcement showed no significant changes in AHR levels and bronchoconstriction after re-exposure to OVA. However, a bronchoconstrictive response, AHR and an increase in inflammatory cells in the bronchoalveolar lavage (BAL) fluid were observed after OVA nebulization exposures following antigen reinforcement [33].

The absence of an obstructive response during the antigenic reinforcement phase could be due to a limited establishment of the inflammatory response. During antigen sensitization, exposure to the allergen occurs in regions distant from the airways. In this context, the primary goal of antigen reinforcement is to direct the initiation of the systemic inflammatory response by inhalation of the allergen directly into the airways. This process allows the previously sensitized inflammatory cells to settle near the airway epithelium and smooth muscle, making them available for activation in response to subsequent antigenic challenges (Fig. 1B) [60].

### Antigenic challenges

Antigenic challenges replicate an exacerbation of allergic asthma as observed in humans after exposure to the specific allergen. In these experiments, the resulting bronchoobstructive response is evaluated after re-exposure to OVA in guinea pigs. In several studies, pretreatment with H1-histamine receptor antagonists such as mepyramine (pyrilamine), chlorphenamine, diphenhydramine or dexamethasone was used to prevent potentially fatal anaphylactic reactions during antigen re-exposure (Table 1). However, in the absence of antihistamines, variability in the nature of the obstructive response has been reported, which also allows the precise recording of the maximal response to OVA in pulmonary function tests, free from interference by external factors unrelated to the allergic response [56]. This approach broadens the scope of the experimental model, as it not only facilitates the assessment of bronchial obstruction but also allows for the evaluation of other relevant pathophysiological processes in allergic asthma, such as the role of pro-inflammatory mediators in immune response exacerbation, smooth muscle contraction, and airway remodeling.

**Table 1:** Main H1-histamine receptor antagonists

Antihistamine	Dose	Pretreatment before the challenge with OVA	Reference
Mepyramine (pyrilamine)	1 mg/Kg	60 min	[61, 62]
	10 mg/Kg	30 min	[63, 64]
	15 mg/Kg	30 min	[65]
	30 mg/Kg		[66, 67]
Chlorphenamine	10 mg/Kg	30 min	[68]
	2 mg/Kg		
Diphenhydramine	20 mg/Kg	60 min	[69]
Dexamethasone	3 mg/Kg		[70]

### Pulmonary function tests in rodents

The techniques used in pulmonary function tests are categorized into invasive and non-invasive procedures. Invasive tests include those that require anesthesia and surgical interventions, such as tracheostomies, intratracheal cannulations, or intrapleural catheterizations [71–73]. These tests allow for the direct measurement of pleural pressure, as well as pulmonary resistance and dynamics, through the evaluation of respiratory flow using a device known as a pneumotachograph. Despite the high precision with which respiratory parameters are obtained from these invasive tests, their application may occasionally compromise the study of various airway diseases, including asthma. This is due to the inevitable use of anesthetics that could directly interfere with relevant signaling mechanisms or pathways in the pathophysiology of asthma, such as xanthines and their derivatives, which induce muscle relaxation mechanisms and could affect model fidelity [74]. Furthermore, the restrictions imposed by controlling "natural" conditions prevent animals from exhibiting conscious or "normal" behaviors, which can result in the loss of environmental interactions and directly affect the biological responses associated with asthma, as emotional components play a crucial role in the response observed in the disease [75, 76].

On the other hand, pulmonary function tests classified as non-invasive, which do not require the use of anesthesia [75], are commonly performed using specialized barometric or volumetric plethysmography chambers. Among these, barometric plethysmography is the most widely used for assessing lung function in various asthma models, including guinea pigs. Despite this advantage, such tests may present reduced data precision, as they are often influenced by spontaneous responses derived from the animals' natural behavior or stress, which may lead to alterations in pulmonary parameters and result in false positives [77]. However, habituating animals to the plethysmography chambers prior to evaluating obstructive responses, along with current technological advancements that have led to the development of specific algorithms in the equipment used, has proven effective in reducing the incidence of false positives associated with these types of responses [33, 78].

### Antigen challenge response

In sensitized guinea pigs, reexposure to the allergen during antigenic challenges triggers a biphasic immune response, consisting of an immediate phase and a late phase [79]. Similarly, in humans, the obstructive allergic response shows considerable variability in its manifestation. It can present as an immediate response, a late response, both, or, in some cases, may not occur at all [28, 80]. In the guinea pig asthma model, it has been observed that the intensity of this response is dependent on the dose of the allergen administered. Lower doses typically induce immediate responses, while higher doses tend to provoke late responses [81].

The immediate allergic response is primarily characterized by the onset of airway obstruction, which occurs minutes after antigen exposure. In the antigen challenge with inhaled OVA, mast cells and basophils are activated, leading to the release of granules containing several mediators. These mediators include histamine (Fig. 1C), a potent stimulator of smooth muscle contraction in the airways, eicosanoids, PAF, enzymes such as tryptase and chymase, and multiple cytokines [82, 83]. These mediators act in various ways on airway smooth muscle and leukocytes (Table 2), collectively causing an increase in vascular permeability, recruitment of inflammatory cells, primarily eosinophils, and an increase in mucus secretion by goblet cells [84, 85]. Together, these processes enhance bronchoconstriction in the allergic asthma model, reducing airway diameter and airflow during bronchospasm [84].

**Table 2:** Inflammatory mediators released during the allergic response in the guinea pig asthma model

*Inflamatory mediator*	*Funcion*	*References*
*IL-4*	Creates an inflammatory environment for the recruitment of inflammatory cells, primarily eosinophils in responses Th2	[86]
*IL-1*	Recruitment of leukocytes such as neutrophils (severe neutrophilic asthma)	[79, 86]
*IL-5*	Increases eosinophilopoiesis	[85]
*TNF-α*	Associated with non-allergic severe inflammation. Recruitment of neutrophils	[86]
*IFN-γ*	Associated with non-allergic severe inflammation. Recruitment of neutrophils	[86]
*IgE*	Activates mast cells and basophils by binding to the FcεR receptor	[87]
*IgG1*	Immunoglobulin predominantly expressed in guinea pigs. Generates a mild obstructive response	[56]
*PAF*	Chemotactic for neutrophils and eosinophils. Enhances mast cell activation and CysLT release. Potentiates histamine-induced bronchoconstriction	[82, 88]
*CysLT*	Binding to its receptor on airway smooth muscle induces contraction. Considered the most potent bronchoconstrictors. Eosinophil chemotaxis, induce plasma extravasation, and cause tissue edema	[85, 86, 88]
*TxA2*	Binding to its specific receptor on smooth muscle causes contraction. Vasoconstriction	[89]
*PGD2*	Eosinophil and Th2 cell chemotaxis	[88]
*Tryptases*	Promote mast cell activation and histamine release. Leukocyte recruitment. Possible link to increased bronchoconstriction through bronchodilator degradation	[90]
*Chymase*	Increases mast cell degranulation	[90]
*Histamine*	Regulates granulocyte accumulation in the airways	[87]
*Eotaxin*	Activation and recruitment of eosinophils	[79]
*Eosinophils*	Release cytotoxic proteins, TxA2, PGD2, CysLT, PAF, oxygen free radicals, and inflammatory cytokines	[86–88]
*Neutrophils*	Activation increases severe neutrophilic asthma, generally non-allergic. Release of PAF	[86, 88]
*Mast cells*	Release histamine, TxA2, PGD2, CysLT, PAF, IL-1, IL-5, IFN-γ, TNF-α. Associated with early responses	[79, 85, 86, 88]

Hours after allergen exposure, following the immediate phase, the late-phase responses begin to manifest. During this phase, lipid mediators such as CysLT, thromboxane A2 (TxA2), PAF, and prostaglandin D2 (PGD2) are produced [56, 86, 91]. PGD2 and TxA2 exert direct effects on airway smooth muscle contraction. PGD2 acts as a potent bronchoconstrictor, while TxA2 has more complex effects, as it is generally associated with airway smooth muscle contraction but can also influence vasodilation. Thus, inflammatory mediators not only affect airway smooth muscle in asthma but also impact the smooth muscle cells of the vasculature [89, 92]. Furthermore, the effects of these mediators act synergistically enhancing the allergic response. For instance, PGD2 and CysLT induce prolonged contraction of the airway smooth muscle, while TxA2 and PAF facilitate the infiltration of inflammatory cells, particularly eosinophils (Fig. 1C) [86].

It is noteworthy that, in guinea pig models subjected to multiple antigenic challenges, in addition to the previously mentioned mediators, there is an increased release of pro-inflammatory cytokines such as IL-1, IL-5, interferon gamma (IFN-γ), TNF-α, and eotaxin, all of which are strongly associated with increased tissue damage in the airways [79, 87]. The cyclic and prolonged release of inflammatory components present in the late phase promotes enhanced infiltration of neutrophils and mast cells into the airways, allowing the observation of several characteristics of chronic asthma in the guinea pig asthma model [19, 28, 29].

The variability in the inflammatory and bronchoconstrictive components present during the immediate and late phases of the allergic response not only allows for the identification of differences in mediator expression and cellular changes between these phases, but also highlights variations associated with the number of antigenic challenges administered to the guinea pigs. This distinction facilitates the classification of disease chronicity levels in the experimental model, enabling a more accurate and comparative evaluation of the mechanisms and characteristics associated with each stage of the disease.

### Levels of chronicity

**Fig. 2:**
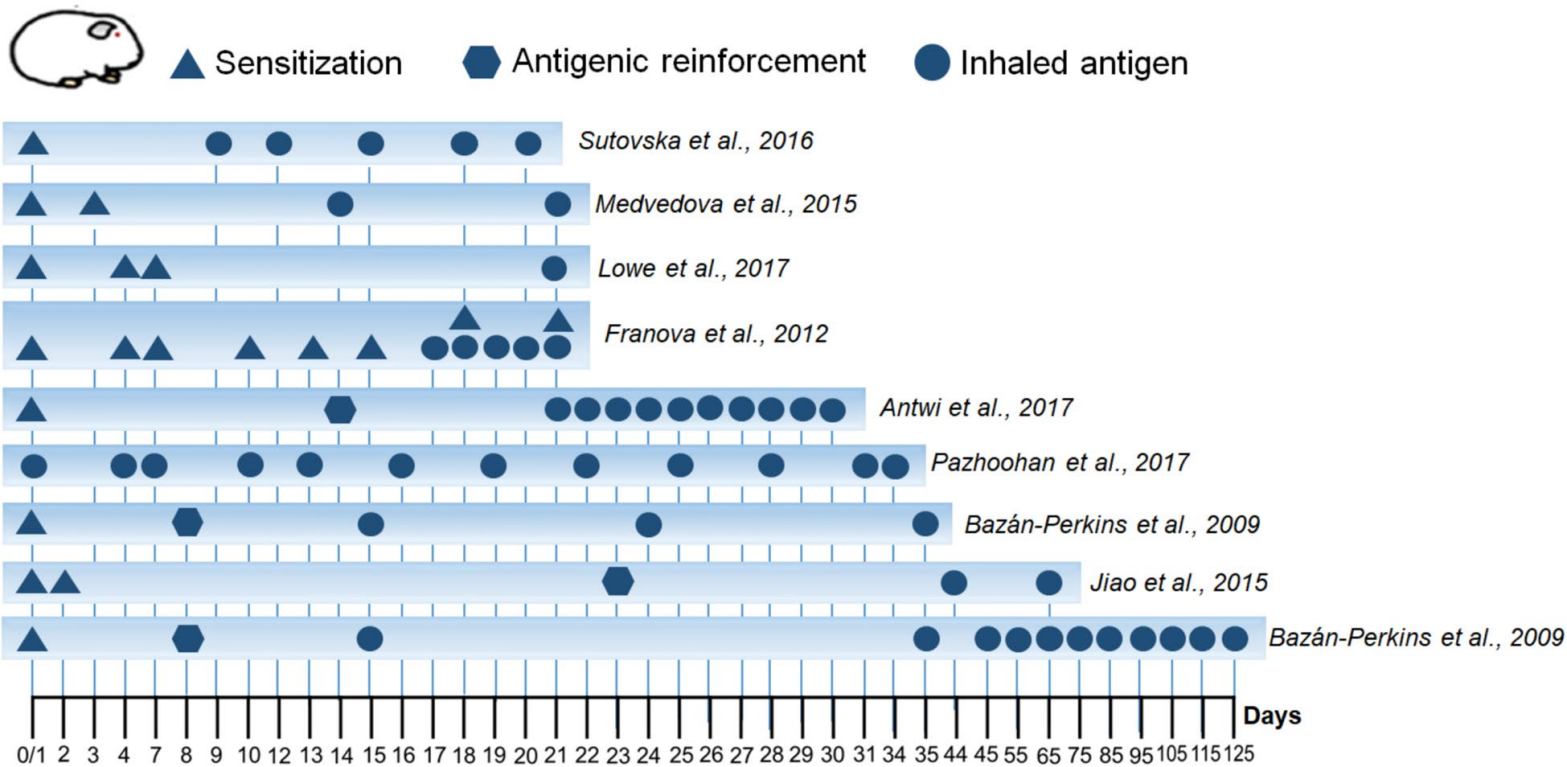
Variability of guinea pig models of allergic asthma. These models are distinguished by different routes of inoculation, administered doses, and the number of antigen challenges, resulting in acute and chronic models. Differences are observed in the presence or absence of allergic sensitization (triangles), antigen reinforcement (pentagons), and antigen challenges (circles). The sensitization phase is crucial for triggering the allergic response, and its variability is attributed to differences in administered doses and routes of administration, with intraperitoneal and subcutaneous injections being the most commonly used (Table 2). Although antigen boosting is a less commonly used phase, some studies consider it essential for evaluating the continuity of allergic and inflammatory responses in the airways

In guinea pig models of allergic asthma, two distinct levels of chronicity can be identified, categorized as acute and chronic models based on the number of antigenic challenges to which the animals are exposed [93, 94]. In the acute model, guinea pigs are typically subjected to three antigenic challenges over an experimental period of approximately 20 to 35 days. In contrast, the chronic model extends for approximately 125 days, during which the animals receive a total of 12 antigenic challenges (Table 3, Fig. 2) [33]. Notably, the duration of the chronic model may vary depending on the experimental protocols employed, as some studies define its onset from day 65 [95].

**Table 3:** Allergic asthma models in guinea pigs

*Model*	*Description*	*Administered dose*
Sutovska et al. 2016 [96]	• Model of 20 days • Sensitization i.p. y s.c. in day 1 and day 3 • Allergen challenges in days 9, 12, 15, 18 and 20 with inhaled OVA	▲ 5 mg OVA + 1 mg/ml Al(OH)_3_/SSF i.p in day 1 ⬤ 5 mg OVA/1 ml SSF s.c. in day 3 Inhaled OVA 1–2 min
Medvedova et al. 2015 [97]	• Model of 21 days • Sensitization i.p. and s.c. in day 1 • Sensitization i.p in day 3 • Inhaled OVA on days 14 and 21	▲ 1% OVA solution in aqua pro inyectione, 0.5 ml i.p. and 0.5 ml s.c on day 1 and 1 ml i.p. in day 3 ⬤ Inhaled OVA by 30 s
Lowe et al. 2017 [26]	• Model of 21 days • Sensitization i.p. on days 1, 4 and 7 • Inhaled OVA in day 21	▲ 150 µg OVA + 100 mg Al(OH)_3_ in 1 ml SSF in sensitization ⬤ 300 µg OVA/1 ml SSF on inhaled OVA
Franova et al. 2012 [98]	• Model of 21 days • Sensitization i.p. and s.c. on day 1 • Repead i.p. OVA inyections every 3 days • Nebulized OVA for the last 5 days	▲ 5 mg OVA + 1 mg Al(OH)_3_ i.p. and 5 mg OVA on 1 ml SSF s.c ⬤ 1% OVA in 0.9% sterile sodium chloride solution for nebulized OVA
Antwi et a. 2017 [70]	• Model of 30 days • Sensitization i.p. on day 1 • Antigenic reforcement i.p. on day 14 • Challenge with inhaled OVA from day 21 to day 30	▲ 2 mg OVA enmulsified in 10 mg ⬣ Al(OH)_3_ dissolved in 10 ml SSF i.p and 1 mg OVA dissolved in SSF i.p ⬤ 1% OVA w/v dissolved in PBS inhaled
Pazhoohan et al. 2017 [99]	• Model of 34 days • Nebulized OVA every 3 days for 5 weeks	⬤ 1 mg OVA/1 ml NaCl 0.9% on days 1,4,7,10 and 13 ⬤ 2.5 mg OVA/1 ml NaCl 0.9% on days 16, 19 and 22 ⬤ 5 mg OVA/1 ml NaCl 0.9% on day 25 and 28 ⬤ 10 mg OVA/1 ml NaCl 0.9% on day 31 and 34 ⬤ All inhaled OVA by 10 min time
Bazán-Perkins et al. 2009 [33]	• Model of 35 days • Sensitization i.p. and s.c. on day 1 • Antigenic reforcement with nebulized OVA on day 8 • Inhaled antigen on days 15, 25, 35 • Model of 125 days • Sensitization i.p. and s.c. on day 1 • Antigenic reforcement with nebulized OVA on day 8 • Inhaled antigen starting from day 15, every ten days until day 125	▲ 0.6 mg/ml OVA + 1 mg/ml Al(OH)_3_ dispersed in SSF ⬣ 3 mg OVA/ml SSF nebulized by 5 min ⬤ From day 15, 1 mg OVA/1 ml SSF and the subsequent challenges, 0.5 mg OVA/1 ml SSF nebulized during 1 min
Jiao et al. 2015 [95]	• Model of 65 days • Cyclophosphamide inyections i.p. on day 1 • Sensitization i.p. on day 2 • Antigenic reforcement i.p. on day 23 • Inhaled OVA on days 44 and 65	▲ Cyclophosphamide 30 mg/kg i.p. 2 mg OVA + 100 mg Al(OH)_3_ i.p ⬣ 0.1 mg OVA + 100 mg Al(OH)_3_ i.p ⬤ 10 mg/ml OVA aerosol for 90 s

i.p.: intraperitoneal, s.c.: subcutaneous

Both acute and chronic guinea pig models of allergic asthma provide distinct insights into disease pathogenesis depending on the chronicity of the condition, allowing researchers to assess different aspects based on the study objectives. The acute model of allergic asthma enables the investigation of allergic inflammation, including Th2 lymphocyte activation mechanisms, their dynamic response, and the increase in inflammatory cells and mediators released during the acute phase of the disease [56, 100, 101]. Additionally, this model facilitates the physiological study of airway smooth muscle bronchoconstriction following antigenic exposure, as well as AHR, providing critical insights into the proteomic regulation of the contractile machinery in allergic asthma [33, 102].

On the other hand, the chronic asthma model is characterized by repeated antigenic challenges and the persistence of late-phase inflammatory events, making it a valuable tool for investigating disease progression in detail. This model allows for the comprehensive analysis of inflammation [70], airway smooth muscle constriction, and AHR, as well as the evaluation of tissue damage and airway remodeling processes [33, 56]. Among the most significant structural alterations observed in this model are goblet cell hyperplasia, epithelial damage, and the accumulation of extracellular matrix proteins, including type I collagen and subepithelial laminin [33, 67, 103]. Collectively, these tissue modifications make the chronic model an essential tool for pharmacological evaluations, as it more accurately mimics the long-term progression of asthma, allowing for a more precise assessment of the therapeutic efficacy of pharmacological treatments [104, 105].

### Evaluation of airway hyperreactivity in allergic asthma guinea pig model

The assessment of AHR is the final characteristic measured in the guinea pig asthma model [28, 33, 56]. The term airway reactivity refers to the normal ability of the airways to contract upon exposure to a contractile stimulus (contractile agonist), while the term hyperreactivity refers to an increase in this contraction capacity. In other words, in AHR, the airways exhibit rapid shortening and high force contraction in response to a minimal contractile stimulus [106].

The study of AHR in guinea pigs is conducted through airway reactivity tests based on responses to contractile agonists such as acetylcholine, methacholine, or histamine. The use of these agonists in AHR analysis is justified by the similarity of the mechanisms regulating smooth muscle contraction in guinea pigs and humans. Furthermore, they have been employed in bronchial provocation tests in asthmatic patients [107].

The mechanism of action of histamine in guinea pig AHR, similar to that in humans, occurs through the signaling pathway of histamine H1 receptors, which activates the phospholipase C (PLC) pathway, leading to the production of 1,4,5-inositol trisphosphate (IP3) and 1,2-diacylglycerol (DAG). This process triggers airway smooth muscle contraction, mediated by an increase in intracellular calcium concentrations and the activation of the actomyosin interaction in the cytoskeleton, through the phosphorylation of myosin light chains induced by myosin light chain kinase (MLCK) [24, 102, 108]. Similarly, acetylcholine and methacholine induce smooth muscle contraction by activating muscarinic M3 receptors, which also signal through the PLC/IP3/DAG pathway. However, unlike methacholine, which is exclusively administered pharmacologically, acetylcholine can be endogenously released by the parasympathetic nervous system [109].

In guinea pigs, exposure to the contractile agonist is typically performed either before or after an antigen challenge, usually with OVA. This approach is essential because, in guinea pigs, AHR only manifests after the release of mediators and cells involved in the allergic inflammatory response, events that are triggered by exposure to the antigenic challenge [28, 33].

In this context, histamine, the most commonly used agonist, can be administered via a single dose before and after the antigen challenge [67, 81] or through dose–response curves with increasing histamine concentrations before and after antigenic challenge [56, 110]. As observed in the studies by Evans [67] and Smith [81], protocols employing a single histamine dose, typically at high concentrations, effectively assess the presence of AHR. However, there are compelling arguments favoring the use of dose–response curves as a more suitable approach for analyzing AHR in guinea pigs. Firstly, histamine has been proposed as a key inducer of systemic anaphylaxis, which can be fatal in animal models when administered at high concentrations [111]. Additionally, a recent study by Álvarez-González [28], which evaluated AHR in 75 guinea pigs using dose–response curves with increasing histamine concentrations, demonstrated that guinea pig responses to histamine varied within a dose range of 0.001–0.13 mg/mL. This finding suggests that using a single dose of histamine may obscure the true response in guinea pigs, potentially leading to inaccuracies in the obtained data [28].

In conjunction with the previously presented findings, the integrated analysis of AHR in the allergic asthma guinea pig model, along with the evaluation of immunological and histological processes, provides a broader perspective on the underlying mechanisms. This approach not only allows for a detailed examination of potential modifications in the smooth muscle of the airways, particularly in relation to the contractile machinery, but also offers a thorough analysis of airway remodeling. Furthermore, it facilitates the identification of potential interactions with other respiratory structures, such as the epithelium and blood vessels, which could play a crucial role in modulating AHR [100]. Such an integrated analysis is essential for understanding the factors contributing to the development and progression of AHR and may provide valuable insights into potential therapeutic targets for the treatment of allergic asthma.

## Conclusions

Allergic asthma is the most prevalent phenotype worldwide, which has led to an increased interest in understanding new aspects of the disease. However, the direct study of asthma in humans is limited by various ethical and practical constraints. In this context, the development of an experimental model of allergic asthma in guinea pigs has not only allowed the emulation of the immunological aspects triggering the disease, but also the replication of many of the pathophysiological features observed in humans, including the variability in responses among individuals within the affected population. This model has facilitated the analysis of various levels of chronicity, both acute and chronic, and has enabled the implementation of protocols incorporating diverse methodological strategies. These strategies include the selection of allergen type, inoculation methods, differences in antigen sensitization approaches, the presence or absence of antigenic reinforcement, the number of antigen challenges, allergen doses administered, variations in assessing hyperreactivity, and model duration. Despite differences in methodological protocols, the guinea pig asthma model reliably identifies the most relevant aspects of the disease, such as the presence of Th2-type inflammatory responses, increased levels of key immunoglobulins like IgE, airway smooth muscle contraction, bronchospasms, hyperreactivity, and airway remodeling. In this regard, the guinea pig allergic asthma model is established as a reliable model that accurately mimics the disease in humans, providing essential information for various scientific studies and contributing to the elucidation of poorly understood cellular and molecular processes related to the development of the disease.

## Data Availability

The data in the present manuscript were collected by searching of literatures as well as involving authors own materials.

## Abbreviations

AHR: Airway hyperreactivity
Al(OH)_3_: Aluminium hydroxide
COVID-19: Coronavirus disease 2019
CysLT: Cysteinyl leukotriene
DCs: Dendritic cells
IFN-γ: Interferon gamma
IgE: Immunoglobulin E
IgG1: Immunoglobulin G1
IL-1: Interleukin 1
IL-13: Interleukin 13
IL-4: Interleukin 4
IL-5: Interleukin 5
MHC-II: Major histocompatibility complex class II
OVA: Ovalbumin
PAF: Platelet activating factor
PGD2: Prostaglandin D2
TCR: T cell receptor
Th0: T naive lymphocyte
Th2: T helper type 2
Thf: Follicular T lymphocyte
TNF-α: Tumor necrosis factor alpha
TxA2: Thromboxane A2
